# Robotic‐Assisted Epiphrenic and Mid‐Oesophageal Diverticulectomy: A Tertiary Centre Experience

**DOI:** 10.1002/rcs.70203

**Published:** 2026-07-11

**Authors:** Federica Cucè, Giovanni Pallabazzer, Mario Antonio Belluomini, Andrea Gennai, Biagio Solito, Paola Marini, Debora Gianetri, Stefano Santi

**Affiliations:** ^1^ General and Upper G.I. Surgery Division, Surgery, Dentistry, Maternity and Infant Department University of Verona Verona Italy; ^2^ Division of Esophageal Surgery Regional Referral Center “Mauro Rossi” for Diagnosis and Treatment of Diseases of Esophagus Azienda Ospedaliero‐Universitaria Pisana (AOUP) Pisa Italy

**Keywords:** diverticulum, esophagus, robotic, surgery

## Abstract

**Background:**

Surgical management of oesophageal pulsion diverticula is advised when symptoms significantly impair quality of life. Robotics can aid in transhiatal dissection in diverticulectomy, but available evidence is limited.

**Methods:**

A retrospective chart review of all consecutive robotic‐assisted oesophageal diverticulectomies with Heller myotomy and Dor fundoplication at a high‐volume centre between 2015 and 2024 was conducted. Surgical technique, patient characteristics, and short‐ and long‐term outcomes were recorded. As this was an observational study with anonymised data, ethical approval was waived.

**Results:**

4/14 patients required abdominal and intrathoracic approaches, and none were converted to open surgery. Trocar‐related abdominal wall bleeding required reintervention; mortality was zero. No leaks or strictures were noted; all reported symptom relief, one reflux and two dysphagia recurrences without strictures or recurrences.

**Conclusions:**

Robotic‐assisted diverticulectomy performed by experienced surgeons appears feasible and associated with acceptable short‐term outcomes. Further prospective studies are needed to confirm long‐term safety and efficacy.

## 1 Introduction

Oesophageal diverticula (ED) are outpouchings composed of mucosa and submucosa herniating through the muscularis of the oesophagus. They are rare entities categorised primarily by anatomic location and aetiology. Epiphrenic diverticula are mainly due to pulsion and occur in the distal third of the oesophagus. The underlying physiopathological mechanism is not fully understood, although it is associated with increased intraesophageal pressure and motility disorders (such as achalasia). The diagnosis of concomitant disorders has been reported to be heterogeneous. This might derive partially from the nonroutine use of high‐resolution manometry and from technical challenges in catheter positioning. When utilising endoscopic guidance for manometric catheter placement to overcome cannulation difficulties, Nehra et al.. reported motility abnormalities in all patients with an oesophageal diverticulum [1]. While epiphrenic diverticula can sometimes present more cranially, in a mid‐oesophageal location, diverticula of the middle oesophagus are most commonly traction diverticula that can occur after mediastinal inflammation or adherences and encompass all oesophageal layers; they are more rarely subject to surgical treatment and were excluded from our sample [2].

Clinical symptoms linked to epiphrenic and mid‐oesophageal pulsion diverticula can be diverse, and they are not directly related to the size of the diverticulum; they can include dysphagia, regurgitation, weight loss, thoracic pain, and, less commonly, bleeding and diverticulitis. Stagnation of food inside the diverticulum is one of the main causes of related symptoms and is more common with narrow neck diverticula. Additionally, understanding which symptoms are caused by the diverticulum and which are due to the underlying oesophageal motility disorder can be difficult [3]. Surgical treatment is usually reserved for more symptomatic patients; historically, it involves thoracotomy or laparotomy [4, 5]. In the past 30 years, many case series have indicated the feasibility of laparoscopic and thoracoscopic diverticulectomy [6, 7, 8].

More recently, robotic‐assisted laparoscopy and thoracoscopy have gained popularity, especially since Endowrist Technology (Intuitive Surgical Corporation, Sunnyvale, CA, US) allows for additional degrees of freedom compared to traditional laparoscopy combined with a fixed high‐definition 3D camera. Easier access to difficult angles is especially beneficial for dissection around the cardia and in the mediastinum through a transhiatal approach, as various case reports describing robotic diverticulectomy have underlined [9, 10].

To date, no large series describing robotic‐assisted oesophageal diverticulum resection through transhiatal and thoracoscopic approaches has been reported. In our institution, robotic oesophageal surgeries for benign and later malignant conditions began in 2012 and the first robotic diverticulectomy was performed in 2015, after a few decades of experience in minimally invasive upper GI surgery, including Heller–Dor procedures [11].

In this study, we aimed to describe all patients who presented with symptomatic pulsion diverticula of the distal two‐thirds of the oesophagus and were treated using robotic‐assisted techniques in our tertiary centre, to assess the feasibility and short‐term outcome of this technique.

## 2 Materials and Methods

We conducted a retrospective cohort study through an electronic patient chart review performed in June 2024 to identify all consecutive robotic‐assisted epiphrenic and midoesophageal pulsion diverticulectomy cases performed at our tertiary referral centre since the first robotic diverticulectomy was performed in 2015. The current study was reported following the Strengthening the Reporting of Observational Studies in Epidemiology (STROBE) 2021 guidelines, and the corresponding checklist can be found in Additional file 1 [12].

### 2.1 Patient Selection

Preoperative evaluations included esophagogastroduodenoscopy (EGDS) and esophagograms for all patients. The patients referred to surgery were those in whom the diverticula were ≥ 3 cm in maximum diameter on esophagogram and demonstrated marked stagnation of contrast medium or ingested food (defined as persistent contrast retention beyond 5 min on timed barium swallow), with or without a concomitant oesophageal motility disorder, regardless of whether a motility disorder was suspected or diagnosed. High‐resolution manometry studies were not feasible in all patients due to diverticula size, in which a blind placement of the catheter is highly challenging and could potentially lead to injury to the thin walls.

Surgery was proposed for patients with large oesophageal diverticula with a pulsion component, accompanied by clinical symptoms compatible with the condition (such as dysphagia, regurgitation, and weight loss) that significantly affected their quality of life. None of the patients presented active diverticular inflammation.

Dysphagia and regurgitation severity were assessed clinically through detailed patient interviews and chart reviews. All patients underwent a comprehensive preoperative evaluation to assess their suitability for minimally invasive robotic surgery, including preoperative cardiopulmonary assessment for a possible thoracoscopic approach, and were thoroughly informed of the procedure's risks and benefits. We discussed in detail with the patients the nature of oesophageal diverticula and the possibility of an underlying major oesophageal disorder, explaining that even without a clear manometric diagnosis, the surgery proposed would be a myotomy and partial fundoplication.

### 2.2 Operative Details

The platform used was the Da Vinci SI HD Surgical System (Intuitive Surgical Corporation, Sunnyvale, CA, US) until March 2016, when the Da Vinci XI HD Surgical System (Intuitive Surgical Corporation, Sunnyvale, CA, US) was introduced. Both the first and assisting surgeons were experienced professionals specialising in upper GI disease who had already performed minimally invasive diverticulectomies and other robotic upper GI surgeries.

Before the beginning and during the surgical intervention, intraoperative esophagoscopy with carbon dioxide insufflation and Rigiflex balloon (Boston Scientific, USA)  placement across the cardia was used to identify the position of the diverticulum and later guide stapler placement, and to allow better identification of circular muscular fibres in the myotomy phase.

**FIGURE 1:**
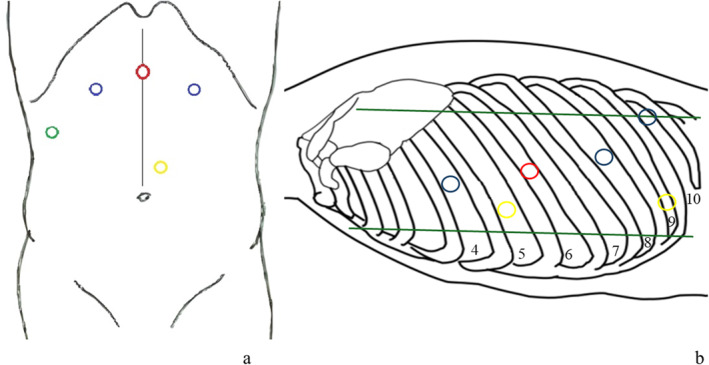
Setup diagram of intraoperative robotic laparoscopic and thoracoscopic trocar positioning: (a) Abdominal approach: red: supraumbilical optic port placement, blue: bilateral midclavicular operative trocars, yellow: AirSeal periumbilical assistant port, green: right costal margin liver retraction and crus traction trocar traction; (b) Thoracoscopic approach: right semiprone decubitus, red: optic ports 6th space, blue: operative ports four 12 mm trocars at 4th and 8th or 10th intercostal spaces, yellow: assistant port at 5th space (or 9th when needed).

In all cases, laparoscopy was employed, and patients were placed in a supine split‐leg position with both arms in adduction. The pneumoperitoneum was achieved using a Veress needle positioned in the middle of the xifo‐umbilical line on the midline; after an intrabdominal pressure of 12 mmHg was reached, the needle was replaced with the optic port. The midclavicular lines were used as landmarks for the two surgical robotic trocars placed symmetrically in the upper quadrants. One airseal assistant periumbilical trocar was inserted below the other trocars for retraction, suction, and stapler use, taking care to maintain an adequate distance to allow for nonconflicted movements. Liver retraction was achieved through a robotic trocar placed 2 cm below the right costal margin on the anterior axillary line, that, when necessary, could also be used for traction on the right diaphragmatic crux. The trocar positioning for the abdominal approach is shown in Figure 1a.

Access to the mediastinum was obtained using a transhiatal approach through accurate crus dissection, while the oesophagus was retracted using a vessel loop tied around the organ. After an adequate dissection around the oesophagus was achieved, preserving the vagal nerves, a linear stapler was placed, isolating the whole neck of the diverticulum. Our aim was to use a single cartridge staple line, although wider diverticula or more angled positioning could not always allow it. The stapler was positioned and fired by an expert assisting surgeon under endoscopic guidance to confirm positioning, adequacy of resection, and absence of perforations or stenosis. After resection and specimen retrieval, the muscular plane above the suture line was closed with a single uninterrupted suture. The diverticulectomy was always followed by a Heller myotomy performed on the anterior oesophageal wall, reaching cranially the same level of the stapler firing and extending at least 3 cm on the stomach wall (reaching in total more than 8 cm of length), aided by endoscopic vision. An air leak test was subsequently performed.

**FIGURE 2:**
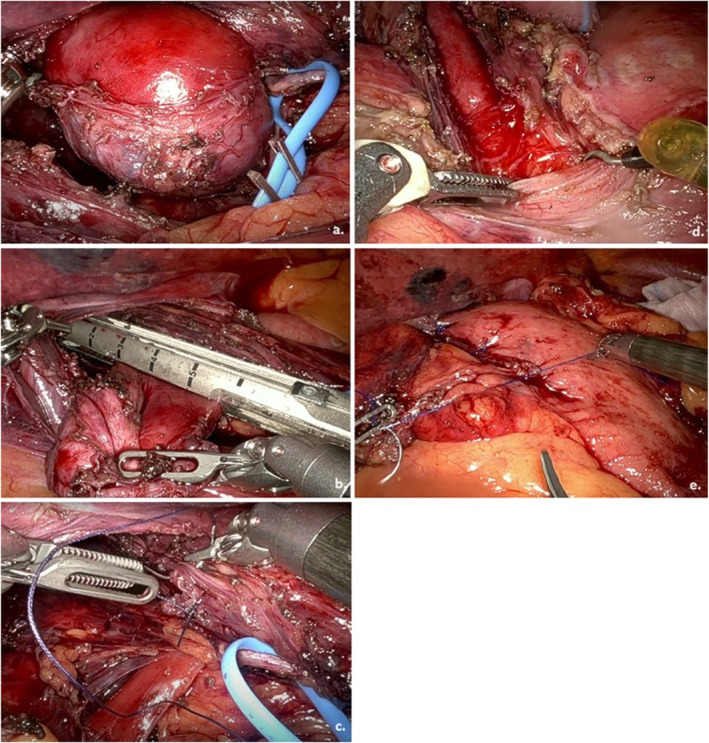
(a) Transhiatal crus dissection, vessel loop oesophageal retraction, vagal nerve preservation; (b) Stapler positioning at diverticulum neck with endoscopic confirmation of placement and resection adequacy; (c) Muscular plane closure with single continuous absorbable suture; (d) Heller myotomy length including at least 3 cm of gastric myotomy, (total > 8 cm); (e) Dor fundoplication anchoring to myotomy edges and diaphragmatic pillars with three interrupted sutures.

The robotic instruments employed were mainly hooks and scissors. To avoid monopolar energy use, haemostasis was achieved by intraesophageal balloon insufflation or through local application of gauze soaked in adrenaline. Dor fundoplication was subsequently performed to prevent postoperative reflux and anchored on both the myotomy edges and the diaphragmatic pillar through three interrupted stitches. The main surgical steps are shown in Figure 2.

The robotic thoracoscopic approach was utilised in addition to robotic laparoscopy for mid‐oesophageal pulsion diverticula with an ample base that could not be reached through transhiatal dissection. The decision to use this approach was based on a combination of preoperative imaging and intraoperative laparoscopic and endoscopic presentation of the diverticulum. A left‐sided thoracoscopic approach was employed because all mid‐oesophageal diverticula in our series that required thoracoscopic access were predominantly protruding towards the left mediastinum, where this laterality provides optimal direct visualisation of the diverticular base; a right‐sided approach may be preferable for higher or rightward‐protruding diverticula, and laterality should be planned accordingly for each case. Thoracoscopic diverticulectomy was performed first, allowing the stapler line to define the cranial extent of the required myotomy. The abdominal robotic phase followed, during which the Heller myotomy was extended from the gastroesophageal junction cranially to the level of the thoracic stapler line and caudally at least 3 cm onto the gastric wall, before completing the Dor fundoplication. Patients were placed in the right semiprone decubitus position to expose the left thorax, four 12‐mm trocars were placed in the 4th, 6th and 8th or 10th intercostal spaces, and an assistant port was placed anteromedially in the 5th space or, when necessary, in the 9th space for retraction, suction, and stapler use. The trocar positioning for the thoracic approach is shown in Figure 1b. After the identification of its base, the diverticulum was skeletonised from surrounding structures, and the stapler was introduced through one of the ports. Its placement, complete firing and adequacy of resection were confirmed through thoracoscopic vision and endoscopy. The specimen was thus retrieved, and the muscular plane was reconstructed surgically.

### 2.3 Postoperative Management and follow up

X‐ray contrast swallow was performed on the third postoperative day (POD) by expert radiologists to exclude oesophageal leakage, stenosis, or altered oesophageal transit. A liquid diet was resumed on the fourth POD, a semiliquid diet on the fifth POD, and a solid diet before discharge. In the case of altered post‐swallow imaging, the diet advancement was delayed, and a personalised protocol was employed.

Outpatient follow‐up was performed through clinical checkups after 4 weeks and every 4 months in the first year. A follow‐up esophagogram was performed within the first 4 months, while endoscopy was performed only if dysphagia, regurgitation, weight loss, or other symptoms arose. Subsequently, an annual follow‐up with an esophagogram was proposed for patients.

### 2.4 Data Collection and Analysis

Data were collected in a retrospective dataset, including demographic characteristics, comorbidities, diverticulum characteristics and dimensions, diagnosis of dysmotility disorders, preoperative symptoms, intraoperative details and technique, length of stay, necessity for intensive care, and postoperative outcomes, including morbidity (classified according to Clavien‒Dindo), mortality and symptoms reported at follow‐up [13]. A digital database was created through Microsoft Excel, and the mean, median, standard deviation, and range were the statistical tools utilised for data reporting. No ethical committee approval was needed as all data were kept and analysed anonymously. Statistical analysis was performed using Microsoft Excel (Microsoft Corporation, Redmond, WA, USA). Given the descriptive and exploratory nature of this retrospective cohort study and the limited sample size, no comparative or inferential statistical analyses were conducted. Continuous variables were summarised using mean, standard deviation (SD), median, and range, according to data distribution and clinical relevance. Categorical variables were reported as absolute frequencies and percentages. Missing data were not imputed; analyses were performed on the available data for each variable, and missing or unavailable assessments were explicitly reported in the Results section. Considering the rarity of the condition and the single‐centre design, the results should be interpreted as descriptive and hypothesis‐generating rather than confirmatory.

## 3 Results

**FIGURE 3:**
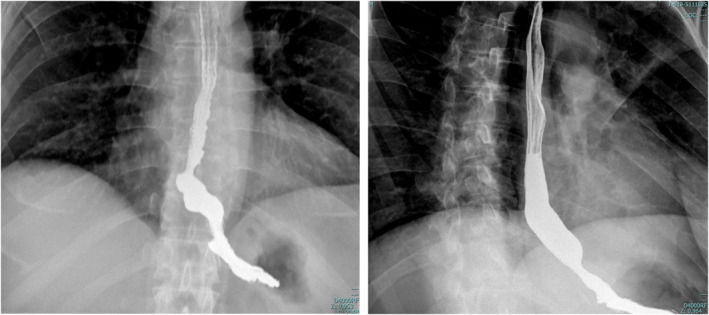
Postoperative oesophagogram showing adequate transit without stenosis or leakage and oesophageal dyskinesia.

The study included 14 patients, 43% of whom were female, with a mean age of 67.9 years (range 46–82 years), 8 (57%) presented with a moderate or severe comorbidity score (categorised according to the Charlson Comorbidity Index score; defined as “moderate” if the score was 3 or 4 points, and a severe when above 5). The mean diverticulum size was 4 cm, two exceeded 5 cm, only in one case accompanied by a diagnosis of major oesophageal motility disorder. The most common symptom was dysphagia followed by regurgitation, while weight loss was rare. Their characteristics are summarised in Table 1. All patients underwent pre‐operative endoscopy and esophagograms. High‐resolution manometry was attempted in 2 out of 14 patients (14%); in the remaining 12 patients, it was not technically feasible due to the size of the diverticulum, which prevented safe catheter advancement without risk of perforation. In these patients, the decision to perform myotomy was standardized: given the association between pulsion diverticula and underlying oesophageal motor dysfunction, Heller myotomy and Dor fundoplication were systematically performed in all cases regardless of manometric confirmation, following the principle that all pulsion diverticula carry a functional component that can benefit from surgical intervention [14]. The patient with achalasia had undergone oesophageal pneumatic dilatation with incomplete resolution of dysphagia. Five (36%) patients had undergone previous abdominal surgery. One case was performed in 2015 with the SI robotic platform, the rest were performed between 2018 and 2024. For four patients, both an abdominal and an intrathoracic approach was required, and none of the cases were converted to open surgery. The linear staplers used in our cohort were SIGNA purple 60 mm cartridges and Echelon Gold or Blue 60 mm cartridges. In this study, 36% of the patients (*n* = 5) required firing of more than one stapler cartridge because of the dimensions or angle of the diverticulum. The mean surgical duration was 353 min, with a range of 210–525 min. Intraoperative adverse events included one case of splenic bleeding, which was controlled with a haemostatic agent application. In three laparoscopic cases, an accidental pleural opening was identified and closed intraoperatively without adverse events. No intraoperative oesophageal perforations were noted. Median estimated blood loss was < 50 mL Incidentally, in two patients, oesophageal dissection revealed oesophageal leiomyomas, which were excised and later confirmed to be benign formations on histological examination. In all cases, the routine postoperative esophagogram was negative for leakage or stricture (Figure 3 shows one of the included patient's postoperative results), and all patients had resumed a soft diet at discharge without adverse events. There was one instance of postoperative vomiting and hypertension (CD = 1) managed conservatively and one case (7%) that required reintervention for trocar‐related severe bleeding (CD = 3b). The mean ICU stay was 1.6 days (range 1–4 days) for the seven patients who required ICU admission. The decision for intensive care unit (ICU) admission was made by a dedicated anaesthesiologist based on patient comorbidities and the duration and complexity of the surgery, as is common practice in our centre after major upper GI surgery. The mean global hospital stay was 7.3 days (median: 7), ranging from 6 to 16 days. At 6 months post‐surgery, all patients reported substantial symptom relief, and none reported regurgitation or dysphagia. At 1 year, one patient reported reflux that was treated with PPIs and two reported dysphagia recurrence, although both reported the symptoms as significantly milder and not as severely limiting in their daily activities compared to their previous condition. They underwent radiological testing, no evident strictures or recurrences were noted in their esophagograms, and no subsequent intervention was needed. The total mean follow up duration was 9.6 months, with a range of 2–15 months; at 6 months, 11 patients (71%) were present at follow up and at 12 months, 4 patients (29%). The perioperative and long‐term mortality rates were zero. The surgical and postoperative outcomes are summarised in Table 2.

**TABLE 1:** Patient demographics and pre‐operative symptoms.

Demographic characteristics	*N* = 14
Age mean—range (y)	67,9 (46–82)
Gender (female)	6 (43%)
Charlson comorbidity index median—range	3 (0–6)
Diverticulum length mean—range (cm)	4 (3–6)
Preoperative symptoms	
Dysphagia	14 (100%)
Regurgitation	7 (50%)
Weight loss	1 (7%)
Major motility disorders diagnosis	1 (7%)

**TABLE 2:** Surgical and postoperative outcomes.

Surgery duration mean—range (min)	353 (210–525)
Transhiatal approach (*n* = 10)	328,5 (210–480)
Combined approach (*n* = 4)	414 (280–525)
Cases of second staple cartridge use	5/14 (36%)
Intraoperative complications	
Bleeding	1/14 (7%)
Accidental pleural opening	3/14 (21%)
Leiomyoma excision	2/14 (14%)
Intensive care unit (ICU) stay	
Patients *N* (%)	7/14 (50%), 1/14 (7%) sub‐intensive care
Length of ICU stay mean—range (days)	1,6 (1–4)
Early postoperative complications (≤ 30 days)	
Oesophageal leak or fistula	0/14 (0%)
Pulmonary complications	0/14 (0%)
C‐D^a^ grade < 3a	1/14 (7%)
C‐D^a^ grade ≥ 3a	1/14 (7%)
Length of hospital stay mean—range (days)	7,36 (6–16)
Long‐term outcomes (follow‐up)	
Dysphagia (mild)	3/14 (21%)
Reflux	1/14 (7%)
Regurgitation	0/14 (0%)
Stenosis	0/14 (0%)
Follow up duration mean—range (months)	9,6 (2–15)

^a^ Clavien–Dindo complication classification.

## 4 Discussion

The clinical manifestations and management of epiphrenic and mid‐oesophageal pulsion diverticula present significant challenges due to their delicate anatomy and frequent association with oesophageal motility disorders. This study's report on minimally invasive surgery for ED adds to the existing body of evidence on the feasibility and satisfactory outcomes of such procedures. Surgery is typically reserved for symptomatic patients or those with large diverticula, considering the nonnegligible morbidity and mortality associated with these procedures. It is usually performed in high‐volume centres, given the rarity of the disease and the delicate and complex anatomy involved. For many rarer conditions, it is advisable to have a dedicated and experienced surgical, endoscopic and radiological team. Only a few centres routinely perform this procedure with robotic assistance. Before performing the first oesophageal robotic diverticulectomy in 2015, in our centre 88 robotic upper GI procedures were successfully performed.

In our retrospective cohort study, we present 14 patients who underwent robotic diverticulectomy, representing to our knowledge, among the largest single‐centre robotic series reported in the current literature. Notably, severe adverse events occurred in only one case, in which the patient underwent reintervention for trocar bleeding, with no occurrence of oesophageal leakage or other oesophageal‐related adverse events. Intraoperative accidental pleural opening did not increase the incidence of pulmonary adverse events (which were absent in our cohort). The decision to transfer the patient to more intensive care after surgery was always preplanned and based on the patient's age or preexisting conditions. In most cases, it was limited to the night of the procedure.

Previous reports on minimally invasive diverticulectomy, such as those by Palanivelu et al.., Fernando et al.., and Boutron et al.., indicated complication rates ranging from 15% to 33%, including a leakage rate ranging from 8% to 20% [14, 15, 16, 17, 18, 19, 20]. Mortality ranged from 0% to 11% [8, 14, 15, 16, 17, 18, 19, 20]. Although our cohort experienced low complication rates compared with previous reports, it is important to emphasise when evaluating this intervention that morbidity and mortality remain considerable, and that risks and benefits should always be discussed with patients thoroughly. The absence of postoperative oesophageal leaks in our cohort may be related to careful patient selection, extensive institutional experience in minimally invasive oesophageal surgery, routine intraoperative endoscopic guidance during stapler positioning, and systematic muscular layer reconstruction after diverticulectomy; however, these findings should be interpreted cautiously given the small sample size, referral‐centre setting, and potential surgeon‐selection bias inherent to this retrospective single‐centre series.

Complete visualisation of the diverticulum neck and its safe dissection from surrounding structures are fundamental for reducing fistulas or strictures. For higher pulsion diverticula that are harder to reach from the abdomen, a combined approach, performed by experienced oesophageal surgeons, can help achieve complete diverticular resection. The choice of adding a thoracoscopic approach should be made considering the dimensions and the distance of the cranial part of the diverticulum opening from the cardia, while evaluating the added risks of this approach considering patients' comorbidities [21, 22]. It should be noted that in our cohort, thoracoscopy increased the average surgical time by approximately 45 min, as should be expected in two‐field surgery requiring patient repositioning and lung exclusion. The two longest surgeries, lasting 510 and 525 minutes respectively, were among those with a combined approach; in the first case, adhesions from a previous surgery were present, whereas in the longest case fibrosis was present around the cardia as the patient had undergone endoscopic dilatation for achalasia.

Accompanying resection with a Heller‐Dor procedure aids in reducing dysphagia due to underlying oesophageal disorders in pulsion diverticula and has been linked to a reduction in oesophageal fistula risk [8].

The accidental discovery of an oesophageal leiomyoma has been previously reported in oesophageal surgeries as it is often undetected preoperatively [23, 24]. When obstructing a clear view and dissection of the oesophagogastric junction, resection can aid surgeons in achieving accurate identification of noble structures and maintaining oesophageal integrity.

In our series, the median hospital stay was 7 days, which is in line with other studies that reported LOSs ranging from 7 to 12 days [6, 17]. Given the delicate and complex nature of the surgical procedure and the potential for adverse events, careful patient selection is fundamental and can greatly influence outcomes across different settings. It is also important to note that to guarantee the best patient selection and care, patients should be referred primarily to centres with substantial experience in minimally invasive oesophageal surgery.

As it is difficult to distinguish symptoms due to mechanical obstacles presented by the diverticulum and underlying oesophageal disorders, defining clinical success of diverticulectomies remains challenging. However, patient satisfaction with the procedure and postoperative quality of life should be the main guide for the surgeon. Clinical success was reported to be approximately 88% in a case series and a review of the literature by Caso et al.. In our series, all patients reported symptoms improvement, with 76% reporting complete remission of dysphagia symptoms [8]. No specific symptom questionnaire is currently validated for oesophageal diverticula and a standard clinical evaluation was employed by the surgeons, that can reduce the applicability of results; nonetheless, in our cohort symptom relief was reported by all patients, none presented with significant dysphagia recurrence during follow up.

The advantages of robotic surgery over conventional minimally invasive oesophageal procedures have been a topic of interest since the widespread diffusion of robotic technology. The possible benefits of the employment of robotics include: (1) enhanced access to the narrow hiatal and mediastinal spaces through the articulation of instruments; (2) the stable, tremor‐filtered camera providing consistent visualisation even in the dynamic thoracic environment during respiration; and (3) the Endowrist technology enabling precise intracorporeal suturing for muscular plane closure and fundoplication [17]. Recent meta‐analyses indicated that among the advantages of newer surgical platforms, there were shorter recovery times, reduced postoperative pain and blood loss for esophagectomies and esophagomyotomies, and accidental oesophageal perforations were significantly reduced. Nonetheless, longer operative times and higher costs, along with limited availability are still significant drawbacks [2, 3, 4, 5, 6, 7, 8, 9, 10, 11, 12, 13, 14, 15, 16, 17, 18, 19, 20, 21, 22, 23, 24, 25, 26, 27, 28]. In our centre, between 2010 and 2015, five standard minimally invasive diverticulectomies were performed (four with a laparoscopic approach and one with a combined laparoscopic and thoracoscopic approach), their operative time ranged between 3 and 4 h, and no significant adverse events were reported. Since 2015, all diverticulectomies have been performed using a robotic approach. This shift reflects the surgical team's perception that the intrinsic features of the robotic platform, such as articulated instruments and enhanced visualisation, may facilitate a more precise and safer dissection and resection of the cranial portion of the diverticulum compared to laparoscopy. However, no comparative studies between laparoscopic and robotic approaches for oesophageal diverticulectomy have been conducted to date, likely due to the rarity of the condition. Any conclusions regarding the relative merits of robotic versus laparoscopic diverticulectomy require properly designed comparative studies that should include direct cost comparison.

A limitation of the current study is its observational design and the retrospective data collection; the small sample precluded subgroup or multivariate analysis. The data were all derived from a single tertiary center, and more extensive analysis with greater numerosity and multicenter recruitment could be beneficial to understand the applicability of the procedure and its results on a wider scale. The reproducibility of the clinical assessment of dysphagia and regurgitation is limited, as the severity of symptoms associated with esophageal diverticula was evaluated by experienced surgeons but without the use of a specific validated clinical scale. Moreover, at long‐term follow‐up, a significant section of participants was missing, undermining the applicability of longer term results.

## 5 Conclusion

Robotic‐assisted diverticulectomy appears technically feasible with encouraging short‐term outcomes for epiphrenic and mid‐oesophageal pulsion diverticula when performed by experienced oesophageal surgeons at high‐volume centres. These results should be interpreted as preliminary evidence of feasibility rather than definitive proof of long‐term safety and efficacy, given the retrospective design, small sample size, and limited follow‐up duration. The abdominal approach allows us to perform an oesophageal myotomy and an antireflux procedure aiming to prevent diverticula and dysphagia recurrence. Experienced oesophageal surgeons can employ a transthoracic approach to resect more cranially placed diverticula with satisfying surgical outcomes. Surgical treatment of oesophageal diverticula could improve patients' reported quality of life, although some may experience symptomatic reflux or residual dysphagia. Further larger prospective or experimental studies are necessary to evaluate the short‐ and long‐term outcomes of robotic approaches compared with other surgical techniques.

## Author Contributions

Dr. Federica Cucè contributed to the data collection, analysis and reporting, Dr. Stefano Santi Dr. Giovanni Pallabazzer, Dr. Mario Antonio Belluomini, Dr. Andrea Gennai, Dr. Biagio Solito, Dr. Paola Marini and Ms. Debora Gianetri contributed to the manuscript conceptualisation and reporting, they all reviewed and approved the final version of the manuscript.

## Funding

The authors have nothing to report.

## Ethics Statement

This was an observational study with anonymised data, and ethical committee approval was waived according to institutional policy for retrospective anonymised analyses.

## Consent

Informed consent was obtained from all the individual participants included in the study. The authors affirm that human research participants provided informed consent for publication of the images in Figure 3.

## Conflicts of Interest

Dr. Stefano Santi has received a nonbinding grant from Olympus. Dr. Federica Cucè, Dr. Giovanni Pallabazzer, Dr. Mario Antonio Belluomini, Dr. Andrea Gennai, Dr. Biagio Solito, Dr. Paola Marini, Ms. Debora Gianetri have no conflicts of interest or financial ties to disclose.

## Supporting information


Supporting Information S1

## Data Availability

The data that support the findings of this study are available on request from the corresponding author. The data are not publicly available due to privacy or ethical restrictions.
